# Supplementary material for: Nucleophile sensitivity of Drosophila TRPA1 underlies light-induced feeding deterrence
Source: eLife. 2016 Sep 22;5:e18425. doi: 10.7554/eLife.18425 (PMC5068967; doi:10.7554/eLife.18425)
Supplement: Supplementary file 1. — DOI: http://dx.doi.org/10.7554/eLife.18425.026 [file elife-18425-supp1.pptx]

## Slide 1
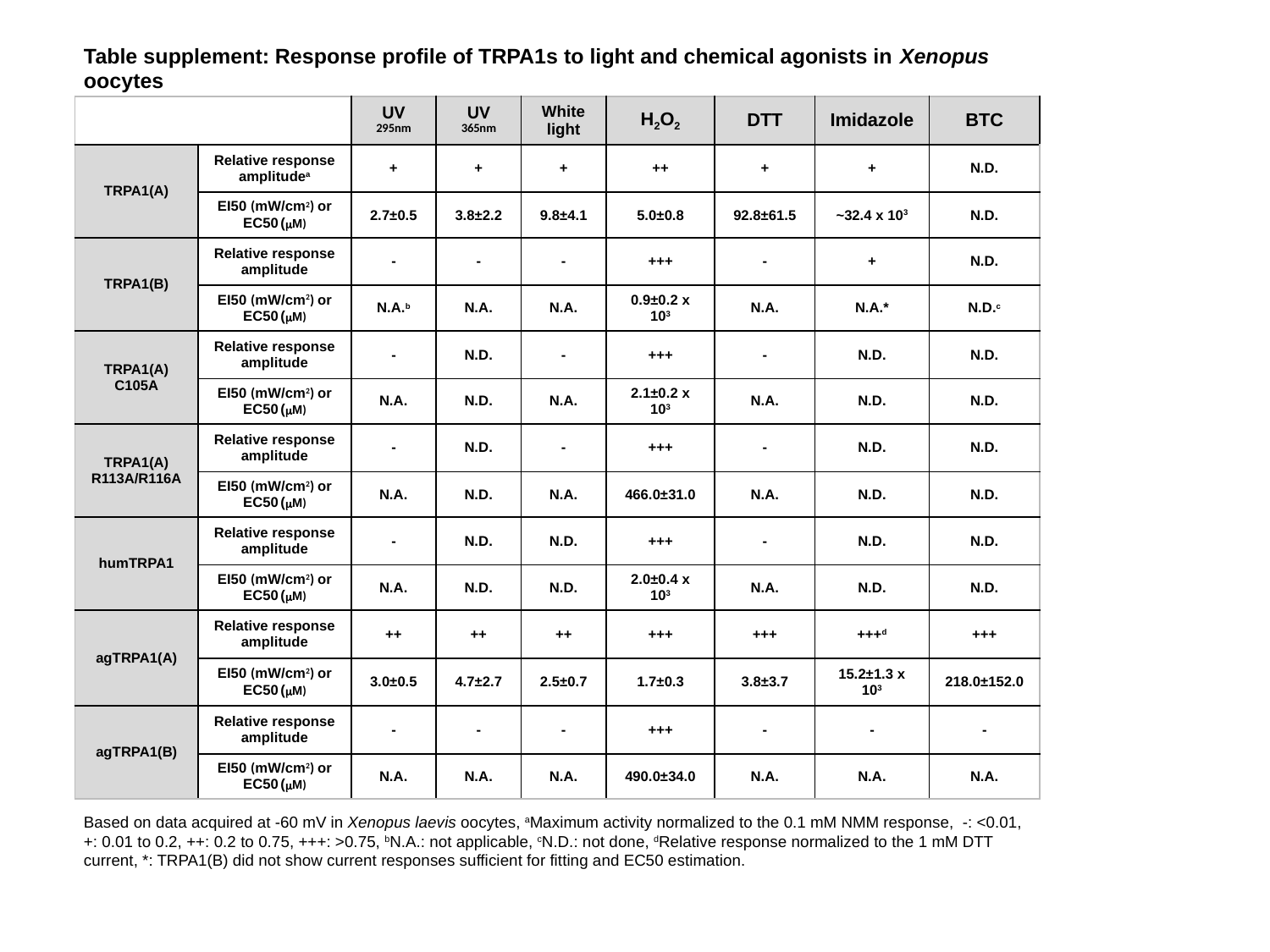

+++ Relative response version
Table supplement: Response profile of TRPA1s to light and chemical agonists in Xenopus oocytes
| | | UV 295nm | UV 365nm | White light | H2O2 | DTT | Imidazole | BTC |
| --- | --- | --- | --- | --- | --- | --- | --- | --- |
| TRPA1(A) | Relative response amplitudea | + | + | + | ++ | + | + | N.D. |
| | EI50 (mW/cm2) or EC50 (mM) | 2.7±0.5 | 3.8±2.2 | 9.8±4.1 | 5.0±0.8 | 92.8±61.5 | ~32.4 x 103 | N.D. |
| TRPA1(B) | Relative response amplitude | - | - | - | +++ | - | + | N.D. |
| | EI50 (mW/cm2) or EC50 (mM) | N.A.b | N.A. | N.A. | 0.9±0.2 x 10^3^ | N.A. | N.A.\* | N.D.c |
| TRPA1(A) C105A | Relative response amplitude | - | N.D. | - | +++ | - | N.D. | N.D. |
| | EI50 (mW/cm2) or EC50 (mM) | N.A. | N.D. | N.A. | 2.1±0.2 x 10^3^ | N.A. | N.D. | N.D. |
| TRPA1(A) R113A/R116A | Relative response amplitude | - | N.D. | - | +++ | - | N.D. | N.D. |
| | EI50 (mW/cm2) or EC50 (mM) | N.A. | N.D. | N.A. | 466.0±31.0 | N.A. | N.D. | N.D. |
| humTRPA1 | Relative response amplitude | - | N.D. | N.D. | +++ | - | N.D. | N.D. |
| | EI50 (mW/cm2) or EC50 (mM) | N.A. | N.D. | N.D. | 2.0±0.4 x 10^3^ | N.A. | N.D. | N.D. |
| agTRPA1(A) | Relative response amplitude | ++ | ++ | ++ | +++ | +++ | +++d | +++ |
| | EI50 (mW/cm2) or EC50 (mM) | 3.0±0.5 | 4.7±2.7 | 2.5±0.7 | 1.7±0.3 | 3.8±3.7 | 15.2±1.3 x 10^3^ | 218.0±152.0 |
| agTRPA1(B) | Relative response amplitude | - | - | - | +++ | - | - | - |
| | EI50 (mW/cm2) or EC50 (mM) | N.A. | N.A. | N.A. | 490.0±34.0 | N.A. | N.A. | N.A. |
NMM 0.1mM 기준
<0.01: -
0.01 to 0.2: +
0.2 to 0.75 : ++
>0.75 : +++
Based on data acquired at -60 mV in Xenopus laevis oocytes, aMaximum activity normalized to the 0.1 mM NMM response, -: <0.01, +: 0.01 to 0.2, ++: 0.2 to 0.75, +++: >0.75, bN.A.: not applicable, cN.D.: not done, dRelative response normalized to the 1 mM DTT current, *: TRPA1(B) did not show current responses sufficient for fitting and EC50 estimation.
